# Assessment of bystander killing-mediated therapy of malignant brain tumors using a multimodal imaging approach

**DOI:** 10.1186/s13287-015-0157-3

**Published:** 2015-09-07

**Authors:** Cindy Leten, Jesse Trekker, Tom Struys, Tom Dresselaers, Rik Gijsbers, Greetje Vande Velde, Ivo Lambrichts, Annemie Van Der Linden, Catherine M. Verfaillie, Uwe Himmelreich

**Affiliations:** Biomedical MRI, Department of Imaging and Pathology, KU Leuven, 3000 Leuven, Belgium; Molecular Small Animal Imaging Center, KU Leuven, 3000 Leuven, Belgium; Imec, Department of Life Science Technology, 3001 Leuven, Belgium; Biomedical Research Institute, Lab of Histology, Hasselt University, 3500 Hasselt, Belgium; Laboratory for Molecular Virology and Gene therapy, KU Leuven, 3000 Leuven, Belgium; Leuven Viral Vector Core, KU Leuven, 3000 Leuven, Belgium; BioImaging Laboratory, University of Antwerp, Campus Drie Eiken, 2610 Antwerpen, Belgium; Department of Development and Regeneration, Stem Cell Institute, KU Leuven, 3000 Leuven, Belgium

## Abstract

**Introduction:**

In this study, we planned to assess if adult stem cell-based suicide gene therapy can efficiently eliminate glioblastoma cells in vivo. We investigated the therapeutic potential of mouse Oct4^−^ bone marrow multipotent adult progenitor cells (mOct4^−^ BM-MAPCs) in a mouse glioblastoma model, guided by multimodal in vivo imaging methods to identify therapeutic windows.

**Methods:**

Magnetic resonance imaging (MRI) of animals, wherein 5 × 10^5^ syngeneic enhanced green fluorescent protein-firefly luciferase-herpes simplex virus thymidine kinase (eGFP-fLuc-HSV-TK) expressing and superparamagnetic iron oxide nanoparticle labeled (1 % or 10 %) mOct4^−^ BM-MAPCs were grafted in glioblastoma (GL261)-bearing animals, showed that labeled mOct4^−^ BM-MAPCs were located in and in close proximity to the tumor. Subsequently, ganciclovir (GCV) treatment was commenced and the fate of both the MAPCs and the tumor were followed by multimodal imaging (MRI and bioluminescence imaging).

**Results:**

In the majority of GCV-treated, but not phosphate-buffered saline-treated animals, a significant difference was found in mOct4^−^ BM-MAPC viability and tumor size at the end of treatment. Noteworthy, in some phosphate-buffered saline-treated animals (33 %), a significant decrease in tumor size was seen compared to sham-operated animals, which could potentially also be caused by a synergistic effect of the immune-modulatory stem cells.

**Conclusions:**

Suicide gene therapy using mOct4^−^ BM-MAPCs as cellular carriers was effective in reducing the tumor size in the majority of the GCV-treated animals leading to a longer progression-free survival compared to sham-operated animals. This treatment could be followed and guided noninvasively in vivo by MRI and bioluminescence imaging. Noninvasive imaging is of particular interest for a rapid and efficient validation of stem cell-based therapeutic approaches for glioblastoma and hereby contributes to a better understanding and optimization of a promising therapeutic approach for glioblastoma patients.

**Electronic supplementary material:**

The online version of this article (doi:10.1186/s13287-015-0157-3) contains supplementary material, which is available to authorized users.

## Introduction

Gliomas arise from glial cells (astrocytes, oligodendrogial and ependymal cells) and are the most common brain tumors in humans. They comprise a broad range of lesions with distinct differences in malignancy, which is classified according to the World Health Organization (WHO) [1]. Glioblastoma multiforme (GBM) are the most malignant tumors (WHO grade IV) in the spectrum of brain tumors [2]. The prognosis of patients diagnosed with GBM is still extremely poor, with a 5-year survival of less than 3 % of patients despite multimodal treatment approaches consisting of surgery and concomitant radio- and chemotherapy [3]. Therefore, new treatment modalities are under investigation, among which is therapy based on the bystander killing effect following suicide gene therapy as has been tested in the past [4–8].

This therapeutic approach relies on administration of cells carrying a suicide gene, such as the gene encoding for the herpes simplex virus-thymidine kinase (HSV-TK). When the substrate for this enzyme is provided, for instance ganciclovir (GCV), it enters the cell and is converted by HSV-TK into GCV-monophosphate. The HSV-TK displays a 1000-fold higher affinity for GCV than the mammalian TK and therefore this targeting approach limits systemic toxicity while the increased affinity boosts tumor therapy capabilities [9]. Subsequently, cellular kinases recognize the GCV-monophosphate and will create GCV-triphosphate, a guanine nucleoside analogue which causes DNA chain termination and subsequent cell death. Due to the formation of gap junctions between adjacent cells, GCV-monophosphate can passively diffuse into neighboring cells which will result in mostly tumor and therapeutic cell killing, as normal adult brain cells usually do not replicate [10]. This is also known as ‘the bystander killing effect’. Previously, attempts were tested in clinical trials to treat glioblastoma patients using viral vectors encoding for HSV-TK by directly introducing the suicide gene into tumor cells, with usually poor results [11]. This is generally believed to be caused by insufficient distribution of the viral vectors throughout the tumor [12]. Therefore, attention turned to carriers such as bacteria [13, 14] and tumor-tracking stem cells [15] to enhance delivery of the suicide gene. Stem cells that are capable of forming gap junctions with infiltrating tumor cells would allow transfer of the GCV-phosphate into neighboring cells, which results in bystander-mediated tumor cell killing [16, 17]. This approach can in theory eliminate both the primary tumor and the infiltrating tumor cells, thus eliminating sources of possible recurrent tumors [9].

The feasibility of this strategy was demonstrated by several groups in both xenograft and syngeneic animal models [5, 6, 9, 18–20]. Miletic et al. [9] and Matuskova et al. [21] demonstrated a potent bystander killing effect of HSV-TK expressing rat bone marrow (BM)-derived tumor-infiltrating progenitor cells on 9 L rat gliomas and human mesenchymal stem cells (hMSCs) on the human glioblastoma cell lines 8-MG-BA, 42-MG-BA and U-118 MG.

In previous reports, different imaging modalities have been used mainly for the assessment of the treatment rather than for guidance of therapy [18]. In a clinical setting, patients are routinely followed up by noninvasive imaging such as magnetic resonance imaging (MRI) and positron emission tomography (PET) [22, 23]. In a preclinical setting, these noninvasive imaging techniques are also available and should therefore, when possible, also be used to guide therapy and evaluate treatment response. Furthermore, additional preclinical imaging modalities such as bioluminescence imaging (BLI) are available to further optimize the acquired information prior to treatment initiation and follow-up of therapeutic efficacy [24]. In addition, molecular imaging methods could also be used to monitor the location of therapeutic cells [25].

We have shown previously that mouse Oct4^−^ BM-derived multipotent adult progenitor cells (mOct4^−^ BM-MAPCs) are safe for in vivo applications and are responsive to GCV in the healthy mouse brain as assessed by BLI [26]. In this study, we intended to assess the therapeutic potential of mOct4^−^ BM-MAPCs in a mouse glioblastoma model and, in particular, the guidance of therapy by cellular imaging methods for optimization of the therapeutic response and for the identification of therapeutic windows. For instance, MRI in combination with superparamagnetic iron oxide (SPIO) particles or BLI in combination with Firefly luciferase (fLuc)-expressing cells can provide information on the location and/or function of cells. The GL261 model was used, which is a mouse glioblastoma model in a syngeneic (immunocompetent) host that has been characterized extensively [27, 28] and is widely considered as the gold standard in glioblastoma research.

## Materials and methods

### Cells

#### Cell culture

mOct4^−^ BM-MAPCs were obtained from the Stem Cell Institute at KU Leuven. Cells were cultured as described previously [29]. In short, dishes coated with fibronectin were used when splitting the cells every 48 hours. Medium contained 60 % low-glucose Dulbecco’s modified Eagle medium (DMEM; Gibco BRL, Sigma-Aldrich, St. Louis, MO, USA), 40 % MCDB-201 (Sigma-Aldrich), 1 × selenium-insulin-transferrin-ethanolamine (ITS; Sigma-Aldrich), 0.2 mg/ml LA-BSA and 0.8 mg/mL powdered bovine serum albumin (BSA; Sigma-Aldrich), 10^−4^ M ascorbic acid 3-phosphate (Sigma-Aldrich), 100 units of penicillin, 1000 units of streptomycin (Gibco®, Invitrogen, Carlsbad, CA, USA), 2 % fetal bovine serum (Gibco®, Invitrogen), 10 ng/mL human platelet-derived growth factor (R&D systems, Minneapolis, MN, USA), 10 ng/mL mouse epidermal growth factor (Sigma-Aldrich), 1000 units/ml mouse leukemia inhibitory factor (Esgro®, Millipore, Billerica, MA, USA) and 1 × chemically defined lipid concentrate (Gibco®, Invitrogen). Finally, β-mercaptoethanol (1 ×; Gibco®, Invitrogen) was added freshly to the media before sterilization with a 22-mm filter (Millipore).

The GL261 cell line, extensively used in mouse models of glioblastoma, was obtained from Dr S. Van Gool, KU Leuven. GL261 cells were cultured as described earlier [30].

#### Transduction

mOct4^−^ BM-MAPCs were consecutively transduced (p24 = 20 pg p24/cell) with two lentiviral vectors with an EF1α promoter [26]. The first transfer plasmid was engineered to express a 3×flag tagged fLuc (3flag-fLuc) together with HSV-TK and a blasticidin resistance cassette (BsdR) linked by a peptide2A sequence (T2A) and an internal ribosomal entry site (IRES), respectively (pCH-EF1a-3flag-fLuc-T2A-HSV-TK-IRES-BsdR). The second transfer plasmid encoded enhanced green fluorescent protein (eGFP) and a puromycin resistance cassette (PuroR), linked by an IRES (pCH-EF1a-eGFP-IRES-PuroR). The lentiviral vectors are referred to as LV_ EF1a-3flag-fLuc-T2A-HSV-TK-IRES-BsdR and LV_EF1a-eGFP-IRES-PuroR, respectively. mOct4^−^ BM-MAPCs were consecutively transduced (p24 = 20 pg p24/cell) with both lentiviral vectors.

First, LV_EF1a-eGFP-IRES-PuroR was used for transduction. Subsequently, cells were selected with puromycin (2–4 μg/ml; Sigma-Aldrich). Following selection, LV_ EF1a-3flag-fLuc-T2A-HSV-TK-IRES-BsdR was used to transduce these cells, which were subsequently selected with blasticidin (20–80 μg/ml; Invivogen, San Diego, USA).

#### In vitro validation of cell labeling with SPIO particles

mOct4^−^ BM-MAPCs were labeled for 24 hours with either Endorem® (Guerbet, Roissy, France) or with in-house-produced SPIO particles (ihSPIO; 20 μg iron/ml) in combination with poly-L-lysin (1.5 μg/ml) (see SMG^2^-mPEGSi nanoparticles in [31] for the properties of the ihSPIO) [31, 32]. Subsequently, cells were washed three times and kept overnight in fresh medium after which 1 × 10^5^ cells were harvested for MRI and 5 × 10^4^ were harvested for induced coupled plasma-optical emission spectroscopy (ICP-OES) measurements (for more details, see also [31, 32]). MRI phantoms containing 500 cells/μl were prepared to validate MRI detectability limits. In vitro T2* MR images were acquired (parameters: multigradient echo pulse sequence, repetition time (TR) = 1500 ms, first echo time (TE) = 4.44 ms with 8 increments of 6.75 ms, matrix size = 400 × 400, in plane resolution = 18.7 μm^2^, slice thickness = 0.35 mm, number of slices = 12).

### In vivo models

#### Stereotactical tumor and/or stem cell injections

All animal experiments were conducted according to the European Union Community Council guidelines and were approved by the Animal Ethics Committee of the KU Leuven. Before surgery, animals were anaesthetized by an intraperitoneal injection with a mixture of ketamine (Ketamine1000, 4.5 mg/kg; Ceva, Pompidou, France)/medetomidin (Domitor®, 0.6 mg/kg; Pfizer, New York, USA). Local analgesia (2 % xylocain; AstraZeneca, London, UK) and antibiotics (6 mg/mouse, Ampiveto-20, 200 mg/ml; VMD, New Haw, Surrey, UK) were administered prior to surgery. After fixation of the animals in a stereotactic frame adapted with a stereotaxic injector (both from Stoelting, Wood Dale, USA), cells were suspended in 5 μl phosphate-buffered saline (PBS) and injected (0.5 μl/min) with a 10 μl Hamilton syringe, equipped with a 22 G needle, into the right striatum of C57Bl6/j mice at the following coordinates: 0.5 mm anterior and 2.0 mm lateral to bregma at 3.0 mm from the dura.

#### In vivo assessment of mOct4^−^ BM-MAPCs SPIO labeling

100 % Endorem®- or ihSPIO-labeled cells were injected stereotactically in the striatum of C57BL6/J mice to assess their detectability in vivo using MRI. For this, PBS, 3 × 10^5^ nonlabeled, 1 × 10^5^ and 3 × 10^5^ Endorem®-labeled, as well as 1 × 10^5^ and 3 × 10^5^ ihSPIO-labeled mOct4^−^ BM-MAPCs, were stereotactically injected (n = 2 for each condition) and monitored using MRI until day 8 postinjection.

#### Glioblastoma mouse model (GL261): suicide gene therapy

GL261 (2.5 × 10^5^) were stereotactically injected in the striatum of C57BL6/j mice and allowed to grow for 2 weeks prior to intratumoral stereotactical injection of 5 × 10^5^ labeled and transduced mOct4^−^ BM-MAPCs [27]. Two weeks after tumor induction, animals were arbitrarily divided into three groups: (1) sham-operated animals, which received a PBS injection, (2) animals that were treated with PBS after receiving 5 × 10^5^ mOct4^−^ BM-MAPCs, and (3) animals that were treated with GCV after receiving 5 × 10^5^mOct4^−^ BM-MAPCs. To reduce the blooming effect on T2*-weighted MRI, which would mask anatomical details in the MRI, only 1 or 10 % of the injected mOct4^−^ BM-MAPCs were labeled with SPIO particles. Weekly MR imaging was performed in addition to imaging before and after mOct4^−^ BM-MAPC injection. BLI was performed following mOct4^−^ BM-MAPC injection. Treatment (GCV (50 mg/kg) or PBS) was administered for 14 consecutive days starting at day 1 post-mOct4^−^ BM-MAPC injection. At the end of PBS/GCV administration, both MRI and BLI were performed to assess tumor response and mOct4^−^ BM-MAPC viability, respectively. Animals responding to treatment were defined as animals with tumors at the end of GCV treatment that were not significantly larger than at the beginning of the treatment (<5 mm^3^). Some animals were followed up after the end of treatment for progression-free survival analysis. Two GCV-treated animals were followed for 184 days without tumor re-growth after which animals were sacrificed. A progression-free survival analysis was performed. For all other experiments, the number of animals used per group is stated in the respective figure legends.

#### Humane endpoints

Animals were sacrificed when symptoms reached grade 3 out of 4 (grade 0 for healthy mice, grade 1 for slight unilateral paralysis, grade 2 for moderate unilateral paralysis and/or beginning hunchback, grade 3 for severe unilateral or bilateral paralysis and pronounced hunchback, and grade 4 for moribund mice) according to [33].

### In vivo imaging

#### MRI

All MR images were acquired with a 9.4 T Biospec small animal MR scanner (Bruker Biospin, Ettlingen, GE) equipped with a horizontal bore magnet and an actively shielded gradient set of 600mT m^−1^ (117 mm inner diameter) using a 7 cm linearly polarized resonator for transmission and an actively decoupled dedicated mouse surface coil for receiving (Rapid Biomedical, Rimpar, Germany). MRI was performed after GL261 tumor induction, prior (day 14 post-glioblastoma injection) and following stem cell injection but before treatment initiation (day 16 post-glioblastoma injection) and at the end of the treatment (day 30 post-glioblastoma injection). Mice were anesthetized with 2 % isoflurane in oxygen for induction prior to scanning and with 1.5 % isoflurane in oxygen for maintenance of anesthesia. Temperature and respiration were monitored throughout the experiment and maintained at 37 °C and 100–120 breaths/minute, respectively. For in vivo tracking of the SPIO-labeled mOct4^−^ BM-MAPCs, three-dimensional T2*-weighted MR images were acquired (parameters: 3D FLASH sequence, TR = 100 ms, TE = 12 ms, flip angle = 20°, isotropic resolution = 78 μm^3^, field of view = 2.0 × 1.5 × 0.75 cm) and analyzed with the Image J software (National Institute of Health, Bethesda, Maryland, USA) using a semi-automated method to calculate the hypointense pixel volume. Furthermore, two-dimensional T2-weighted MR images (parameters: axial orientation, RARE sequence, TR = 3157.6 ms, TE = 48.8 ms, matrix size = 256 × 256, FOV = 2.5 × 2.5 cm, in plane resolution = 78 μm^2^, number of slices = 24, slice thickness = 0.5 mm) and MRI scans in coronal orientation (parameters: RARE sequence, TR = 3000 ms, TE = 50.2 ms, matrix size = 256 × 256, FOV = 2.5 × 2.5 cm, number of slices = 16, slice thickness = 0.5 mm, in plane resolution = 78 μm^2^) were acquired to follow-up the tumor size. The area of the lesion was determined by outlining it manually on all slices using the Paravision software (Version 5.1, Bruker Biospin). The sum of the cross-sectional area was used to determine the total tumor size.

#### BLI

Mice were anesthetized with isoflurane (2 % induction, 1.5 % maintenance) in oxygen and placed in the IVIS® 100 imaging system (Perkin Elmer, Waltham, MA, USA). The body temperature was maintained at 37 °C throughout the experiment. D-Luciferin (126 mg/kg in PBS; Promega) was injected intravenously and bioluminescent images were acquired. Data were analyzed for maximum intensity of the photon flux by the living image® 2.50.1 software (Perkin Elmer).

#### Histology

Animals were sacrificed by an intraperitoneal overdose of nembutal (300 μl; Ceva). They were subsequently perfused with a 4 % ice-cold paraformaldehyde (PFA) solution in PBS (Sigma-Aldrich). After overnight post-fixation in 4 % PFA, the brain tissue was stored in a 0.1 % sodium azide solution in PBS (Fluka; Sigma-Aldrich) at 4 °C. Paraffin sections (5 μm) were sliced and a Masson trichrome, a Prussian blue staining and an Iba1 staining were performed [31]. For the latter, sections were stained with an Iba1 antibody (1/250). Visualization was achieved using the Dako EnVision + system-HRP (DAB) kit (Dako, Glostrup, Denmark).

#### Statistical analysis

Statistical analysis was performed using GraphPad Prism (GraphPad Software PRISM, La Jolla, CA, USA). Significant differences between GCV- and PBS-treated animals regarding total tumor volumes (MRI; mm^3^) and cell viability (BLI; P/s) were determined by means of an analysis of variance test with differences of *p* < 0.05 regarded significant. Figures show mean ± standard error of the mean (SEM).

## Results

### In vitro assessment of SPIO labeling of eGFP-fLuc-HSV-TK-expressing mOct4^−^ BM-MAPCs

**Fig. 1:**
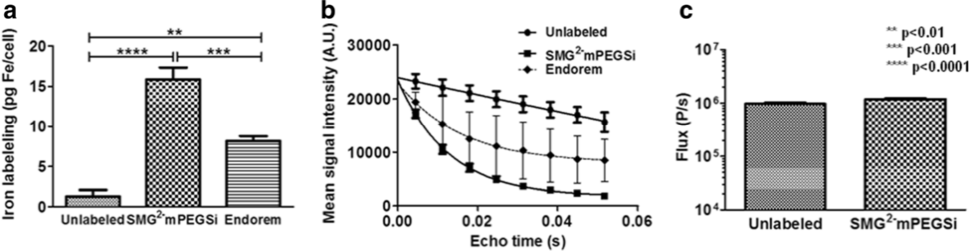
In vitro validation of SPIO labeling of mOct4^−^ BM-MAPCs. **a** ICP-OES data showed superior labeling of mOct4^−^ BM-MAPCs using ihSPIO particles compared to labeling using Endorem® with unlabeled cells containing 1.3 ± 0.8 pg Fe/cell, ihSPIO-labeled mOct4- BM-MAPCs containing 15.9 ± 1.5 pg Fe/cell and Endorem®-labeled mOct4^−^ BM-MAPCs 8.2 ± 0.6 pg Fe/cell. **b** An MRI phantom confirmed these results and showed the corresponding in vitro contrast generation caused by the presence of iron in the cells with enhanced signal decay corresponding to higher concentrations of iron/cell. **c** BLI using D-Luciferin as a substrate indicated no marked differences in cell viability between labeled and unlabeled cells. *SMG* ^*2*^ *-mPEGSI* second seed mediated growth-mPolyethylene glycol

eGFP-fLuc-HSV-TK-expressing mOct4^−^ BM-MAPCs [26] were labeled with ihSPIO particles [31] or Endorem® after which characterization was performed using ICP-OES, MRI and BLI (Fig. 1). ICP-OES (Fig. 1a) showed an iron uptake of 15.9 ± 1.5 pg iron/cell for ihSPIO-labeled cells compared to 8.2 ± 0.6 pg iron/cell for Endorem®-labeled cells. Using optimized labeling conditions, in vitro cell labeling with ihSPIO-labeled cells showed high contrast (low signal intensity) on MRI (Fig. 1b) and no marked differences in cell viability when comparing labeled and unlabeled cells as assessed by BLI (Fig. 1c). Considering that only 1–2 pg iron are needed for cell imaging, these results indicate that, for both contrast agents, single cell visualization in vitro is in principle feasible and safe [34].

### In vivo assessment of mOct4^−^ BM-MAPCs SPIO labeling

**Fig. 2:**
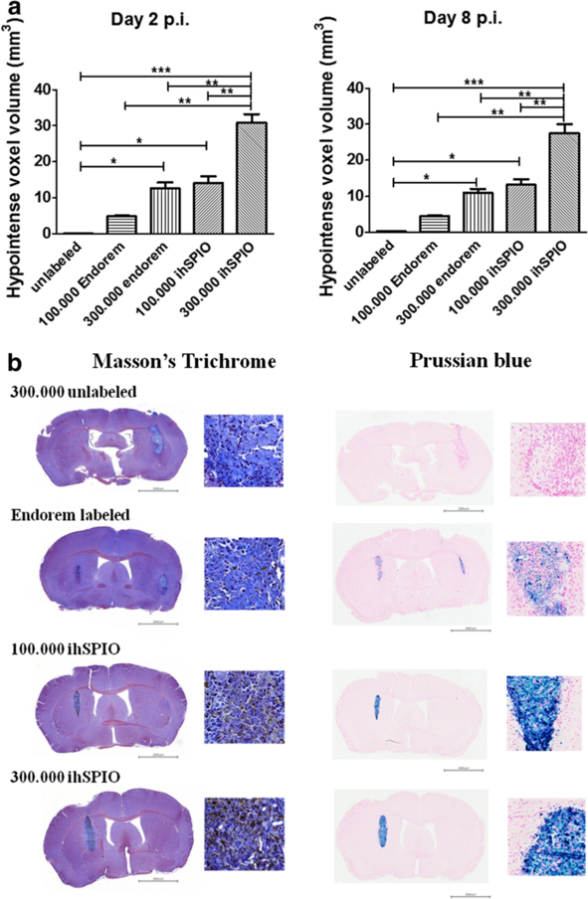
In vivo assessment of mOct4^−^ BM-MAPCs SPIO labeling. **a** Analysis of in vivo three-dimensional T2* MR images on day 2 and day 8 postinjection showed hypointense voxel volume of the Endorem®- and ihSPIO-labeled mOct4^−^ BM-MAPCs. **b** MRI data corresponded to the Masson’s trichrome staining to locate the stem cells and a Prussian blue staining to stain for the presence of iron in the tissue. *p < 0.05, **p < 0.01, ***p < 0.001, ****p < 0.0001. *ihSPIO* in-house superparamagnetic iron oxide, *p.i.* postinjection

Next, we compared the contrast generated by mOct4^−^ BM-MAPCs labeled with ihSPIO particles or Endorem® in vivo. For this, 3 × 10^5^ nonlabeled, 1 × 10^5^ and 3 × 10^5^ Endorem®-labeled, as well as 1 × 10^5^ and 3 × 10^5^ ihSPIO-labeled mOct4^−^ BM-MAPCs were injected into the striatum of C57BL6/j mice after which MRI was performed to assess in vivo contrast generation. Contrast could be detected in vivo on day 2 and day 8 (Fig. 2a) postinjection, with only a slight decrease in contrast from day 2 to day 8 as indicated in Fig. 2a. Three hundred thousand ihSPIO-labeled cells generated a more extensive hypointense contrast compared to mOct4^−^ BM-MAPCs that were labeled with the same amount of Endorem®, indicating that contrast generation with ihSPIO was superior for in vivo cell detection. Masson’s Trichrome and Prussian blue stainings were performed on paraffin sections to confirm the presence of the SPIO particles. The Prussian blue staining confirmed the presence of iron (Fig. 2b) in areas corresponding to the location of hypointense signal in MRI. Both contrast agents generate sufficient contrast to visualize the location of engrafted cells for at least 1 week. Due to its superior imaging properties, cells were labeled with ihSPIO for all subsequent studies.

### In vivo suicide gene therapy using eGFP-fLuc-HSV-TK (+), ihSPIO-labeled mOct4^−^ BM-MAPCs in a mouse glioblastoma model

**Fig. 3:**
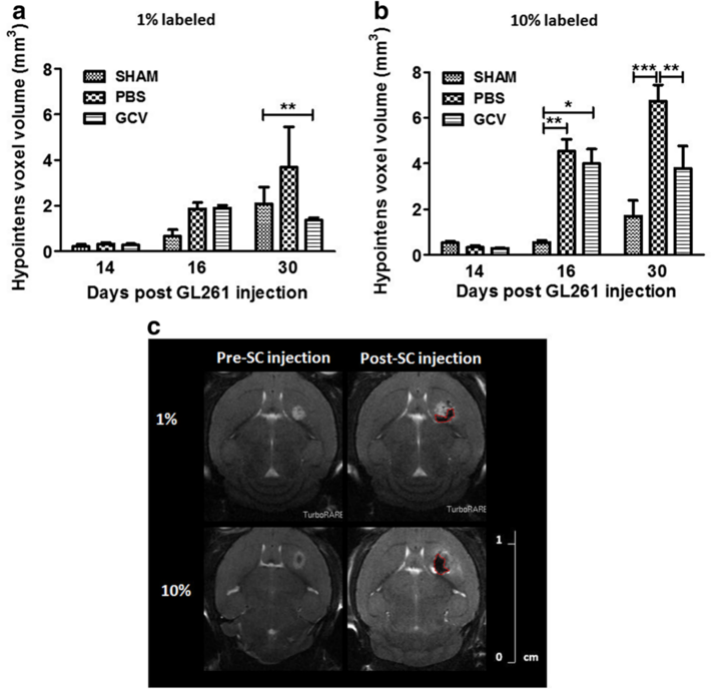
In vivo suicide gene therapy using eGFP-fLuc-HSV-TK (+), ihSPIO-labeled mOct4^−^ BM-MAPCs in a mouse glioblastoma model. **a** Hypointense signal volume determination (three-dimensional T2* MRI) on 1 % labeled mOct4^−^ BM-MAPCs did not show statistically significant differences between stem cell injected animals (phosphate-buffered saline (PBS)/ganciclovir (GCV)) and sham-operated animals (SHAM) 1 day after surgery (day 16). At the end of treatment (day 30), there was, however, a significant difference between sham-operated animals and GCV-treated animals (**p < 0.01) due to the smaller tumor sizes in the GCV-treated group. In the sham-operated and PBS-treated group, tumor formation was present which is accompanied with necrosis and bleedings when tumors grow over time. In contrast, the hypointense signal in the GCV-treated group, which did not develop tumors, is mostly generated by remaining labeled mOct4^−^ BM-MAPCs. **b** Hypointense signal volume determination (three-dimensional T2* MRI) on 10 % labeled mOct4^−^ BM-MAPCs showed statistically significant differences between stem cell injected animals (PBS/GCV) and sham-operated animals 1 day after surgery (day 16). At the end of treatment (day 30), there was a statistically significant difference between sham-operated and PBS-treated animals and between the PBS-treated and GCV-treated groups. *p < 0.05, **p < 0.01, ***p < 0.001. **c** MR images of one representative animal stereotactically injected with 1 % or 10 % labeled mOct4^−^ BM-MAPCs showed that the hypointense contrast, delineated in red, generated by 10 % labeled mOct4^−^ BM-MAPCs was more pronounced compared to 1 % labeled cells. *SC* stem cell

We have previously shown that HSV-TK containing mOct4^−^ BM-MAPCs can be killed in vitro using GCV concentrations as low as 0.01 μM [26]. Furthermore, we have also shown that mOct4^−^ BM-MAPCs could be killed successfully in vivo following treatment with GCV (14 days, 50 mg/kg) [26]. In this study, we assessed whether administration of GCV to mice with HSV-TK containing mOct4^−^ BM-MAPCs present in and around a glioblastoma (GL261) could also kill the glioblastoma cells via a suicide killing bystander effect [9]. Hereby, it is essential to assess the capacity of eGFP-fLuc-HSV-TK-expressing and ihSPIO-labeled mOct4^−^ BM-MAPCs to distribute within and around the glioblastoma. We have engrafted 5 × 10^5^ mOct4^−^ BM-MAPCs into the mouse brain, of which only a fraction (1 or 10 %) of the injected cells was labeled with ihSPIO particles, as 3 × 10^5^ cells already generated extensive hypointense contrast (Fig. 3). Evaluation of the grafted cells by MRI showed that when 10 % (Fig. 3b), but not 1 % (Fig. 3a) of the stem cells were labeled with ihSPIO particles it was possible to follow them by MRI as indicated by the hypointense signal. One representative animal is shown in Fig. 3c.

**Fig. 4:**
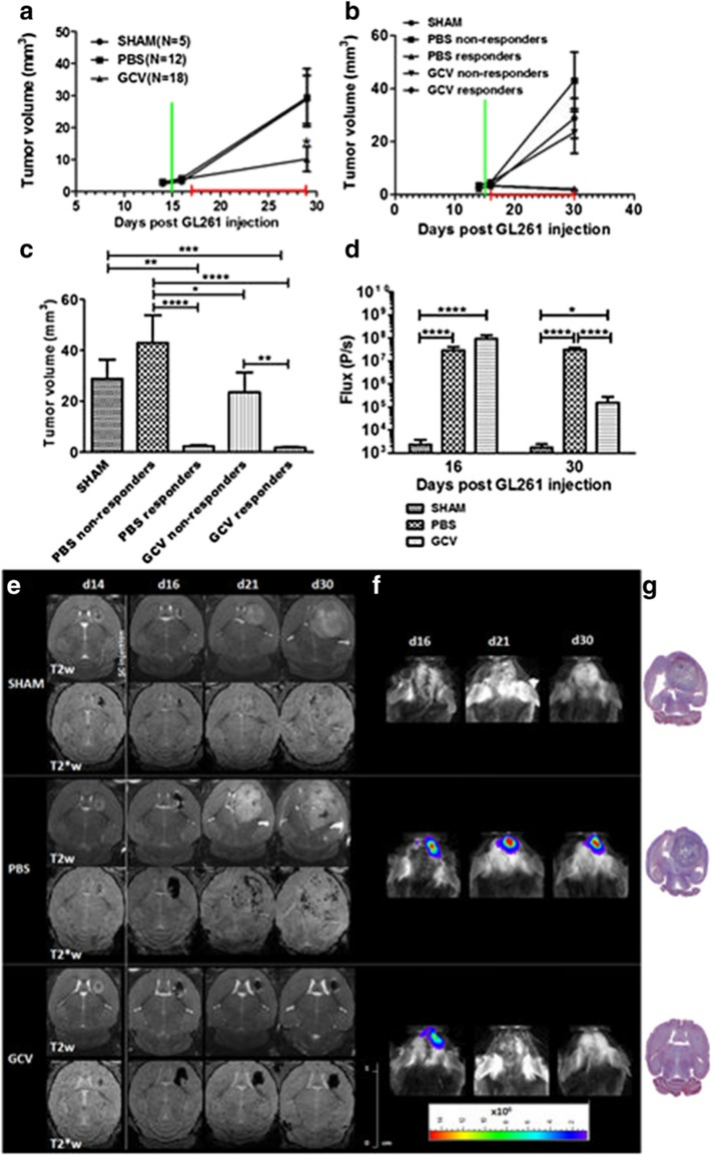
MRI and BLI images of one representative animal for each group. **a** Animals from the ganciclovir (GCV)-treated group displayed significantly smaller tumors at the end of GCV treatment compared to phosphate-buffered saline (PBS)-treated and sham-operated (SHAM) animals (*p < 0.05). Some substantial variability was noticed, however, between individual animals so subgroup analysis was performed (green bar: mOct4- BM-MAPC steretactical injection; red bar: treatment phase). **b** Representation of subgroup tumor development over time for sham-operated, PBS- and GCV-treated animals. No statistically significant differences could be found on day 14 and 16 between the different groups (green bar: mOct4- BM-MAPC steretactical injection; red bar: treatment phase). **c** Statistical analysis of the tumor volumes at the end of treatment (day 30) showed a statistically significant difference in tumor size between GCV responding (n = 11) animals and sham-operated (n = 5)/PBS-treated animals (n = 8). Furthermore, some GCV-treated animals (n = 7) did not respond to therapy whereas some PBS-treated (n = 4) animals also showed a reduced tumor size at the end of treatment. *p < 0.05, **p < 0.01, ***p < 0.001, ****p < 0.0001. **d** mOct4^−^ BM-MAPC viability measurements showed a reduced cell viability for the GCV but not the PBS treated group. *p < 0.05, ****p < 0.0001. **e** MR images of animals of different groups show a comparable tumor growth prior to mOct4^−^ BM-MAPC injection on T2-weighted coronal MR images (upper row for each group) whereas there is little hypointense contrast visible on three-dimensional T2* MR images. In sham-operated animals this mild hypointense contrast was maintained as tumors grew larger although some increase in the hypointense voxel volume, due to the development of necrosis and bleedings, was observed. mOct4^−^ BM-MAPC-injected animals (PBS and GCV) could be detected by three-dimensional T2* MRI (lower row for each group) on day 1 after injection. For animals which developed tumors, the hypointense voxels got more dispersed over time as tumors grew whereas mice responding to GCV treatment had little tumors where labeled mOct4^−^ BM-MAPCs could still be detected at the end of GCV treatment. **f** BLI measurements showed a persistence of the BLI signal in PBS-treated animals indicating survival of the mOct4^−^ BM-MAPCs whereas GCV-treated animals showed a decreased viability after GCV treatment. Sham operated animals were used as negative controls. **g** Histological overview images of brain sections from the respective animals of each treatment group stained with trichrome staining. *d* Day

Stereotactical injection of 5x10^5^ mOct4^−^ BM-MAPCs, of which 10 % were ihSPIO labeled, into the striatum of GL261 tumor bearing C57BL6/j mice was performed. For this, GL261 tumors were allowed to expand for 14 days after which MRI was performed to ascertain tumor growth (Fig. 4 =d14). Subsequently, stem cells were stereotactically injected and MRI and BLI were performed on the following day (=d16) to ascertain stem cell location in and around the tumor. PBS or GCV (50 mg/kg) administration commenced on day 16 and continued for 14 consecutive days (i.e. until d30 following GL261 tumor induction). At d30 post GL261 injection, sham operated animals and some PBS treated animals started to develop grade 3 symptoms that were related to tumor growth, and needed to be sacrificed. MRI and BLI were performed weekly for the duration of the experiment for the assessment of tumor growth and stem cell viability.

Figure 4e (lower rows) confirmed that 10 % labeled stem cells could be tracked by MRI due to their ihSPIO labeling.

Using T2*-weighted MR images, we were able to demonstrate for all animals receiving 10 % labeled mOct4^−^ BM-MAPCs that the tumors contained and were surrounded by ihSPIO-labeled mOct4^−^ BM-MAPCs from the first imaging time point post-engraftment (day 16 postinjection). Tumor volumes were determined from T2-weighted MR images (Fig. 4e, lower row). Tumors were present in all three groups of animals.

When analyzed on a group level, GCV-treated animals showed significantly smaller tumors after the end of treatment when compared to sham and PBS-treated animals (Fig. 4a) However, we noticed some substantial variability between individual animals.

While all sham-operated animals (n = 5) showed substantial tumor masses at the end of the treatment period (day 30), some of the PBS-treated animals showed a substantially smaller tumor size compared to the rest (n = 4/12 PBS-treated animals; Fig. 4b and c). Among the GCV-treated animals, all animals showed apparent delay in tumor development. Fifteen out of 18 animals showed a significantly reduced tumor volume when compared to sham and PBS-treated animals (<15 mm^3^). When considering our predefined criteria of ‘no tumor growth’ compared to the start of treatment (volume <5 mm^3^), 11 animals responded to treatment while 7 animals did show at least some increase in tumor volume (Fig. 4b and c). MRI analysis showed that sham-operated (n = 5), nonresponding PBS-treated animals (n = 8) and GCV-treated, nonresponding animals (n = 7) developed tumors on day 30 after transplantation of the glioblastoma cells that were not statistically different in size (Fig. 4b and c). For all GCV-treated animals, BLI signal intensity decreased significantly (Fig. 4d and f). No difference in BLI signal intensity was seen between responding and nonresponding animals within the GCV- and PBS-treated groups (data not shown). Furthermore, a few animals injected with mOct4^−^ BM-MAPCs and treated with PBS also displayed a reduced tumor size (n = 4) at the end of PBS administration (Fig. 4b and c), although the BLI signal from the mOct4^−^ BM-MAPCs did not change over time (Fig. 4d and f). Imaging results were confirmed by histology (Fig. 4g and Additional file 1: Figure S1).

**Fig. 5:**
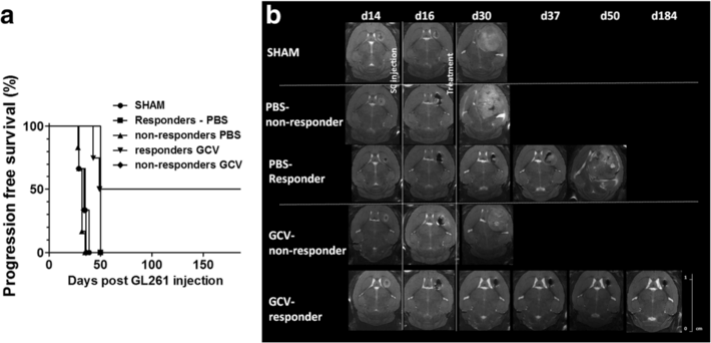
Progression-free survival analysis. **a** A number of animals were observed for a progression-free survival analysis. Sham-operated animals (n = 3; SHAM), phosphate-buffered saline (PBS)-treated (n = 6) and ganciclovir (GCV) (n = 3) unresponsive animals had comparable progression-free survival curves whereas the animals that responded to GCV (n = 4) treatment and one animal treated with PBS but with a reduced tumor size after treatment displayed a prolonged progression-free survival with two animals of the GCV-treated group surviving for 184 days without any sign of tumor re-growth. **b** T2-weighted MRI images showing tumor growth evolution in one representative animal for each of the three treatment groups. *d* Day, *SC* stem cell

**Fig. 6:**
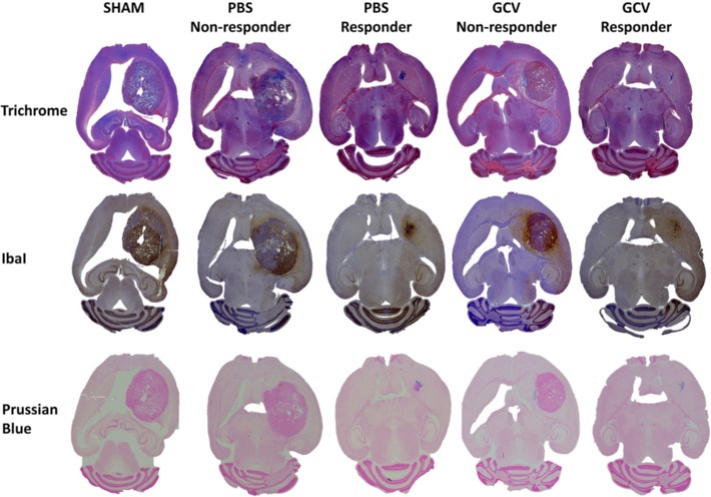
Histological analysis of representative animals at the end of treatment. Animals were sacrificed at the end of treatment and brain sections were stained using Massson’s Trichrome staining (*top*) for overall histological assessment, Iba1 staining (*middle*) for assessment of macrophage activation inside and surrounding the tumor, and Prussian blue staining (*bottom*) as an iron staining of brain sections from all different treatment groups, including examples from responding and nonresponding animals. See Additional file 1 (Figure S1) for further high-magnification images and a more elaborate explanation regarding the observations made based on these staining. *GCV* ganciclovir, *PBS* phosphate-buffered saline, *SHAM* sham-operated

A number of animals were observed long term to determine progression-free survival (Fig. 5). Progression-free survival curves generated for sham-operated animals (n = 3) and PBS-treated animals (n = 6) were comparable. From the GCV-treated animals, some (n = 3) did not respond to treatment and showed a similar survival to the sham-operated and PBS-treated animals. However, some GCV-treated (n = 4) and one animal treated with PBS displayed a longer progression-free survival, with two animals of the GCV-treated group surviving long term (184 days) without any signs of tumor re-growth (Fig. 5a). The median progression-free survival of sham-operated animals (35 days) was significantly lower compared to GCV responding animals (119.5 days) (*p* = 0.0101). Furthermore, histological analysis was performed to confirm the data obtained in vivo. Masson’s trichrome staining showed the presence of large blood vessels explaining the hypointense signal in large tumors. Prussian blue staining confirmed the presence of ihSPIO particles in and around the tumor most likely due to the engrafted mOct4^−^BM-MAPCs. In small lesions, as seen in the therapy responders, iron staining was more concentrated around the injection site in comparison to large tumors as seen in nonresponders. Finally, Iba I staining for the presence of activated microglia was performed which showed remarkably few activated microglial cells inside the tumor, although microglial activation was more pronounced surrounding the tumor (Fig. 6 and Additional file 1: Figure S1). Quantitative comparison between the different groups based on Iba I staining was difficult due to the reduced lesion size in responding animals and apparently higher cell densities (Additional file 1: Figure S1).

In summary, longitudinal in vivo MRI and BLI monitoring confirmed that HSV-TK-expressing mOct4^−^ BM-MAPCs are able to suppress tumor growth after administration of GCV in the majority of the animals. Unexpectedly, a small number of PBS-treated animals that received mOct4^−^ BM-MAPCs also showed reduced tumor sizes.

## Discussion

Although efforts have been made to ameliorate the prognosis for glioblastoma patients, results have been limited so far [35]; the translation of preclinical research to clinical applications has been especially difficult. In the past years, suicide gene therapy has been investigated as a new therapeutic approach [8]. Unfortunately, results obtained in clinical trials have been disappointing, mainly due to insufficient expression and poor intratumoral distribution of the viral vectors encoding for the suicide gene [36]. Therefore, HSV-TK-expressing stem cells that are able to track tumor cells [4–7, 9] have been considered as vehicles for suicide gene delivery. Increasing emphasis on noninvasive follow-up aims to better understand the fate of stem cells in the preclinical phase [25, 37] with the ultimate aim of better translatability from mouse to man. Tumor size, viability and location can be validated with MRI, PET or BLI prior to stem cell injection, thus identifying outliers earlier [25, 37]. Furthermore, the location and viability of the stem cells prior to initiation of treatment is crucial as this will greatly influence the outcome of bystander suicide therapy. Moreover, individual animals can be followed longitudinally, thus reducing the number of animals needed, while additional information can be obtained to better understand the dynamics of experimental glioblastoma treatment approaches. Outliers that are based on experimental errors such as failure of correct cell implantation can also be identified.

In this study, GL261 tumor bearing C57BL6/j mice received 5 × 10^5^ syngeneic eGFP-fLuc-HSV-TK-expressing and ihSPIO-labeled (1 % or 10 %) mOct4^−^ BM-MAPCs for longitudinal follow-up of suicide gene therapy in immunocompetent animals following in vitro and in vivo feasibility studies for cell detection.

Hypointense signal volume determination (three-dimensional T2* MRI) on 1 % labeled mOct4^−^ BM-MAPCs did not show statistically significant differences between stem cell-injected animals (PBS/GCV) and sham-operated animals 1 day after surgery (day 16). However, at the end of treatment (day 30), there was a significant difference between sham-operated animals and GCV-treated animals due to the smaller tumor sizes in the GCV-treated group. In the sham-operated and PBS-treated group, tumor formation was present which was accompanied with necrosis and bleedings when tumors grew over time. In contrast, the hypointense MRI signal in the GCV-treated group, which did not develop tumors, was mostly generated by ihSPIO label, which remained present in the brain after killing of the mOct4^−^ BM-MAPCs.

MRI data showed that labeled mOct4^−^ BM-MAPCs were located in and in close proximity to the tumor from 1 day postinjection for all animals. Therefore, treatment was commenced on day 1 postinjection following multimodal imaging (MRI/BLI). In the majority of GCV-treated, but not PBS-treated, animals a significant difference was found in mOct4^−^ BM-MAPC viability and tumor size at the end of treatment (Fig. 3). While MRI confirmed the location of labeled mOct4^−^ BM-MAPCs around the tumor lesion for all animals, three GCV-treated animals did not show delayed tumor growth for unknown reasons. However, not only the majority of GCV-treated animals but also some PBS-treated animals showed a significant decrease in tumor size compared to sham-operated animals. Although no direct experimental evidence could be provided in our study, one hypothesis in the suicide gene therapy field is that the initial, limited intratumoral killing of both stem cells and tumor cells, caused by injection of the stem cells and the inhospitable tumor microenvironment, generates the release of immunostimulatory molecules [38–40]. This may contribute to overcome immune suppression by the tumor. Our results show, however, that all PBS-treated animals displayed mOct4^−^ BM-MAPC survival during PBS administration as determined by BLI. This indicates that the observed effect on tumor growth in the PBS-treated group is probably not majorly influenced by mOct4^−^ BM-MAPC cell death. Another possible explanation for the reduction of tumor growth could be the immunomodulatory properties of MAPCs themselves and their capability to exert both immune suppression and immune stimulation [41], which might cause the immune system to favor immune stimulation rather than immune suppression in the tumor setting and thus contribute to a therapeutic effect and a reduction in tumor size. As this goes beyond the scope of our study, further research is required to confirm this hypothesis. Independent of the possible immune stimulation of mOct4^−^BM-MAPC engraftment, our studies demonstrate clearly that adding GCV treatment augments killing of the glioblastoma, which is likely the result of the typical bystander killing effect, and could possibly be enhanced by additional immune-stimulation. Furthermore, histological analysis showed the presence of large blood vessels explaining the hypointense signal in large tumors (Masson’s Trichrome) and the presence of ihSPIO particles in and around the tumor (Prussian blue staining). In smaller tumors, iron staining was more concentrated to the injection site in comparison to large tumors. Microglial cells were identified mostly at the tumor border, although areas of microglial activation within the tumor tissue were also found. However, no clear differences could be found between sham-operated, PBS- and GCV-treated animals in microglial activation status, which again stresses the need for future studies focusing on immunological aspect of this treatment model.

## Conclusions

We have shown that suicide gene therapy using mOct4^−^ BM-MAPCs as cellular carriers is effective in reducing the tumor size in the majority of the GCV-treated animals, resulting in a longer progression-free survival compared to sham-operated animals. The treatment can be guided and followed in vivo by MRI and BLI. Hereby, MRI can be used to ascertain stem cell location prior to treatment initiation and follow-up of tumor size. BLI can be used to assess stem cell viability prior to and following treatment. Thus, outliers can be detected earlier, GCV treatment can be initiated based on stem cell distribution rather than on empirical time points, and a more thorough follow-up can be provided prior to and following treatment of these animals. This will contribute to a rapid and efficient validation of stem cell-based therapeutic approaches for glioblastoma and therefore to a better understanding and optimization of a promising therapeutic approach for glioblastoma patients.

## Additional file

Additional file 1: Figure S1. High magnification images from brain sections of representative animals for all groups and subgroups. Massson’s Trichrome staining (*top*) of sham-operated and PBS-injected, control animals showed very large tumors with some bleeding. Prussian blue (*bottom*) staining was also performed, which indicated presence of iron (*) in PBS-injected, control animals that did not respond to treatment. For these animals, positive iron staining was seen both at the tumor border (left Prussian blue staining) and inside the tumor mass (right Prussian blue staining). GCV-treated responders showed high iron content as the cells remained more localized around the much smaller tumor lesions. The presence of high amounts of iron is an indication for the presence of SPIO from engrafted, labeled stem cells. Finally, Iba1 staining (*middle*) was performed which showed microglial activation in the GCV-treated responding group. Microglial activation was also pronounced around the tumor in sham-operated and PBS-injected, control animals. In general, Prussian blue and Iba1 staining appears more intense in animals that responded to treatment due to the small (former) lesion site. (TIFF 6465 kb)

## Abbreviations

BLI: Bioluminescence imaging
BM: Bone marrow
BSA: Bovine serum albumin
BsdR: Blasticidin resistance cassette
DMEM: Dulbecco’s modified Eagle medium
EF1α: Elongation factor 1α
eGFP: Enhanced green fluorescent protein
fLuc: Firefly luciferase
GBM: Glioblastoma multiforme
GCV: Ganciclovir
hMSC: Human mesenchymal stem cell
HSV: Herpes simplex virus
ICP-OES: Induced coupled plasma-optical emission spectroscopy
ih: In-house
IRES: Internal ribosomal entry site
LV: Lentiviral vector
MAPC: Multipotent adult progenitor cell
mOct4^−^: Mouse Oct4^−^
MRI: Magnetic resonance imaging
PBS: Phosphate-buffered saline
PET: Positron emission tomography
PFA: Paraformaldehyde
PuroR: Puromycin resistance cassette
RARE: Rapid Acquisition with Relaxation Enhancement
SEM: Standard error of the mean
SMG^2^-mPEGSI: Second seed mediated growth-mPolyethylene glycol
SPIO: Superparamagnetic iron oxide
T: Tesla
T2A: Peptide2A sequence
TE: Echo time
TK: Thymidine kinase
TR: Repetition time
WHO: World Health Organization
