# Supplementary figures and images for: Assessment of bystander killing-mediated therapy of malignant brain tumors using a multimodal imaging approach
Source: Stem Cell Res Ther. 2015 Sep 7;6(1):163. doi: 10.1186/s13287-015-0157-3 (PMC4562202; doi:10.1186/s13287-015-0157-3)

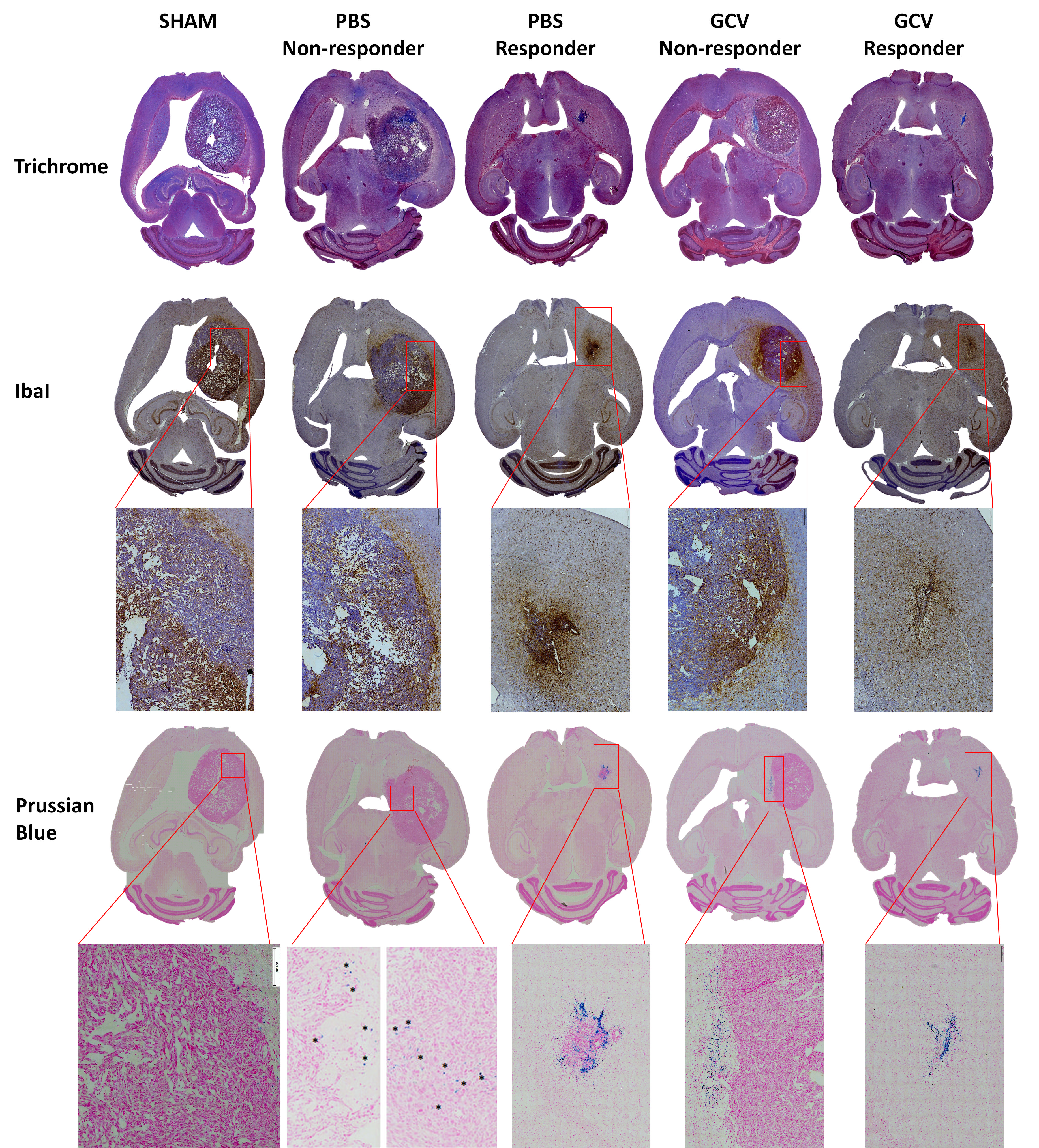

Supplement: Additional file 1: Figure S1. — High magnification images from brain sections of representative animals for all groups and subgroups. Massson’s Trichrome staining (top) of sham-operated and PBS-injected, control animals showed very large tumors with some bleeding. Prussian blue (bottom) staining was also performed, which indicated presence of iron (*) in PBS-injected, control animals that did not respond to treatment. For these animals, positive iron staining was seen both at the tumor border (left Prussian blue staining) and inside the tumor mass (right Prussian blue staining). GCV-treated responders showed high iron content as the cells remained more localized around the much smaller tumor lesions. The presence of high amounts of iron is an indication for the presence of SPIO from engrafted, labeled stem cells. Finally, Iba1 staining (middle) was performed which showed microglial activation in the GCV-treated responding group. Microglial activation was also pronounced around the tumor in sham-operated and PBS-injected, control animals. In general, Prussian blue and Iba1 staining appears more intense in animals that responded to treatment due to the small (former) lesion site. (TIFF 6465 kb) [file 13287_2015_157_MOESM1_ESM.tiff]
